# Targeting Homocysteine and Hydrogen Sulfide Balance as Future Therapeutics in Cancer Treatment

**DOI:** 10.3390/antiox12081520

**Published:** 2023-07-29

**Authors:** Avisek Majumder

**Affiliations:** Department of Medicine, University of California, San Francisco, CA 94143, USA; avisek.biochem@gmail.com

**Keywords:** targeted therapy, cancer biology, hyperhomocysteinemia, gene–environment interaction, epigenetics, stress response

## Abstract

A high level of homocysteine (Hcy) is associated with oxidative/ER stress, apoptosis, and impairment of angiogenesis, whereas hydrogen sulfide (H_2_S) has been found to reverse this condition. Recent studies have shown that cancer cells need to produce a high level of endogenous H_2_S to maintain cell proliferation, growth, viability, and migration. However, any novel mechanism that targets this balance of Hcy and H_2_S production has yet to be discovered or exploited. Cells require homocysteine metabolism via the methionine cycle for nucleotide synthesis, methylation, and reductive metabolism, and this pathway supports the high proliferative rate of cancer cells. Although the methionine cycle favors cancer cells for their survival and growth, this metabolism produces a massive amount of toxic Hcy that somehow cancer cells handle very well. Recently, research showed specific pathways important for balancing the antioxidative defense through H_2_S production in cancer cells. This review discusses the relationship between Hcy metabolism and the antiapoptotic, antioxidative, anti-inflammatory, and angiogenic effects of H_2_S in different cancer types. It also summarizes the historical understanding of targeting antioxidative defense systems, angiogenesis, and other protective mechanisms of cancer cells and the role of H_2_S production in the genesis, progression, and metastasis of cancer. This review defines a nexus of diet and precision medicine in targeting the delicate antioxidative system of cancer and explores possible future therapeutics that could exploit the Hcy and H_2_S balance.

## 1. Introduction

**Figure 1:**
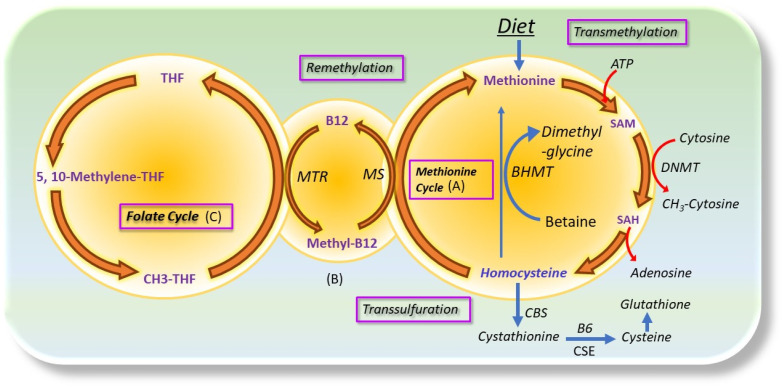
Schematic diagram of Hcy production through the methionine and folate cycle. (**A**) Dietary methionine is converted to homocysteine (Hcy) through S-adenosyl methionine (SAM) and S-adenosyl homocysteine (SAH) and then back to methionine (MET) via the remethylation pathway. Half of Hcy goes to the transsulfuration pathway, where it is converted to cysteine with the help of cystathionine-β synthase (CBS) and cystathionine-γ lyase (CSE). Then cysteine is further converted to glutathione (GSH); (**B**) Conversion of cobalamin (vitamin B12) to methyl-B12 in the presence of methionine synthase reductase (MTR) is necessary for remethylation of 5-methyl-tetrahydrofolate (THF) to THF; (**C**) Dietary folic acid (vitamin B9) enters the folate cycle after its conversion first to dihydrofolate (DHF) and then to THF. The 5, 10-methyltetrahydrofolate reductase (MTHFR) is a key enzyme that converts 5, 10-methylene-THF to 5-methyl-THF [15].

Cancer is the second leading cause of death after cardiovascular disease [1]. Current understanding characterizes cancer into six hallmarks: maintaining proliferative signaling, bypassing growth suppressors, resisting apoptosis, enabling replicative immortality, inducing angiogenesis, and initiating invasion and metastasis [2]. Due to the high proliferative rate, cancer cells depend on many nutrient sources from the diet [3]. Methionine is one of the nutrients that cancer cells require to maintain cell proliferation, growth, survival, and metastasis [4]. Methionine was the first amino acid used in protein synthesis in the eucaryotic system [5]. As an essential amino acid, methionine is not produced in our bodies, so it must be consumed from the diet [6]. Methionine is not only essential for the formation of all proteins, but it also provides lots of other metabolites that are required in multiple other metabolic processes [7]. Via the methionine cycle (Figure 1), methionine can be converted to S-Adenosyl methionine (SAM), the only methyl group donor in DNA, RNA, and histone methylation reactions. DNA, RNA, and histone methylation are dynamic; these regulate gene expression and alter cellular signaling [8]. After transferring methyl group, SAM converts to S-Adenosyl homocysteine (SAH), which then converts to homocysteine (Hcy) [7]. Hcy is a sulfur-containing nonproteinogenic amino acid; after production, half of the Hcy goes to the transsulfuration pathway to produce cysteine (a semi-essential amino acid), and another half of the Hcy can be remethylated back into methionine with the help of the folate cycle [7]. Cysteine is a semi-essential amino acid obtained from the diet or by de novo synthesis from the methionine cycle [9]. In the transsulfuration pathway, when cysteine is produced, other than helping in protein formation, it is also used for hydrogen sulfide (H_2_S) and glutathione (GSH) production [7]. Due to the high proliferative rate, cancer cells mainly depend on the methionine cycle for methylation reaction as well as the production of H_2_S and GSH [10]. A study found that cancer cells express high levels of methionine transporter SLC43A2 for the consumption of more methionine, which causes cancer progression [11]. Many studies found that a methionine restriction diet can reduce cancer risk and progression through various molecular processes [10,12,13]. A phase 1 trial also showed that it is tolerable for metastatic cancer patients to be on a methionine-restricted diet to reduce tumor growth [14].

Cancer cells depend highly on the methionine cycle, producing a massive amount of Hcy [16]. As high levels of intracellular Hcy can be secreted to blood, so many studies correlate high Hcy levels with cancer [17,18,19]. More than 15 μmol/L of Hcy in the blood is clinically termed hyperhomocysteinemia (HHcy) [7]. HHcy has been associated with multiple disease conditions, including cancer [20]. Elevated levels of Hcy are connected with oxidative stress, ER stress, apoptosis, protein oxidation, inflammation, and impaired angiogenesis [17,20]. Moreover, many previous studies found variable associations of polymorphisms of Hcy metabolism genes with cancer [21,22,23], suggesting the possible role of gene–environment interactions in the causation of cancer [24]. Some studies suggested that together with genetic polymorphisms, dietary methionine, folate, vitamin B12, B6, and alcohol consumption play an essential role in the genesis of tumors [25,26,27]. Also, different studies showed that specific genetic polymorphisms may induce risk for specific cancer types [28,29]. More studies are needed on a large number of patients in order to understand which genetic polymorphisms predispose to which types of cancer and how lifestyle modifications could be helpful in reducing cancer risk.

Cancer cells depend on the methionine cycle for their cellular turnover, producing toxic Hcy [20]. Cancer patients show high Hcy levels, but that does not mean that high Hcy is a risk factor for cancer, rather that cancer cells shuttle more Hcy to the transsulfuration pathway [20]. As high Hcy levels lead to many cellular pathogeneses, cancer cells transfer excess Hcy to the transsulfuration pathway for the production of H_2_S [20]. Recent studies revealed that cancer cells increased the expression of CBS (the rate-limiting enzyme in transsulfuration reaction) to reduce excess Hcy levels and produce H_2_S [30]. Previously, H_2_S was considered a toxic gas; however, recent research found that H_2_S has beneficial effects in reversing cellular pathophysiology [7]. H_2_S emerged as a third gasotransmitter after NO and CO [31], and it has been shown to have beneficial effects in reducing oxidative and ER stress, apoptosis, and inflammation, and improving neoangiogenesis [17]. Studies in colon and ovarian cancer mainly showed that this higher production of H_2_S induced tumor growth via inducing cell proliferation and angiogenesis [30]. Also, suppressing CBS expression led to a reduction in tumor growth [32]. This suggests that cancer cells maintain a balance of H_2_S and Hcy levels for their cellular growth and metastasis. Very limited studies have exploited this delicate balance of H_2_S and Hcy as a therapeutic opportunity for cancer treatment. Many studies showed that H_2_S reverses all the pathophysiological effects of Hcy [17]. Many antifolate drugs and drugs targeting Hcy metabolism have long been used to treat cancer; however, all showed limited clinical efficacy due to multiple reasons [19]. In the future, more research is needed that exploits the Hcy and H_2_S balance to treat cancer patients. This review article summarizes the Hcy metabolism and how Hcy metabolism and H_2_S production are associated with cancer. This review also discusses the current therapeutics and future therapeutic opportunities that target these pathways in cancer treatment.

## 2. Homocysteine Production and Hyperhomocysteinemia

**Figure 2:**
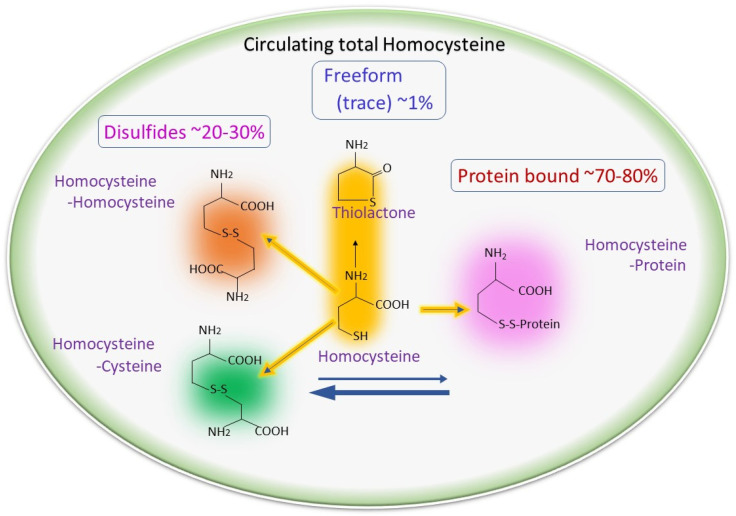
Different forms of homocysteine found in blood circulation: Hcy can be secreted from the cells and can be detected in blood in three different forms: around 1% as free thiol, 70–80% present bound with plasma proteins, and the remaining 20–30% present as homo/heterodimerized with other thiols.

As discussed in the above section, after production, half of the Hcy goes to the transsulfuration pathway, and the other half of the Hcy remethylates back to methionine with the help of the folate cycle [33,34,35]. As Hcy can be secreted into the blood, so different forms of Hcy can be found in blood circulation, as shown in Figure 2 [36]. In the transsulfuration pathway, Hcy is converted to cystathionine with the help of cystathionine β-synthase (CBS), where vitamin B6 (pyridoxine) is an essential co-factor [8]. This is the rate-limiting step of the transsulfuration pathway [8]. After production, cystathionine is further converted to cysteine by cystathionine γ-lyase (CSE), and this cysteine further produces GSH [37].

In normal conditions, cells maintain a delicate balance of Hcy production (through the methionine cycle) and elimination of Hcy (via the transsulfuration pathway) [37]. The normal range of plasma Hcy levels for young adults (~30 years) is 4.6–8.1 μM, and for older adults (30 years and above) is 4–15 μM [38]. In different disease conditions, the balance between the production and elimination of Hcy becomes affected [39,40]. High levels of Hcy in the blood circulation are called hyperhomocysteinemia (HHcy), a systemic disorder [7]. Patients with HHcy show more than 15 uM plasma Hcy [41]. HHcy has been classified as moderate (15–30 μM), intermediate (30–100 μM), and severe (>100 μM) [41]. Genetic mutations in the CBS and methylenetetrahydrofolate reductase (MTHFR) genes (involved in the folate cycle) can lead to HHcy [42,43,44,45]. Different genetic variants of the CBS and MTHFR genes that lead to HHcy can also be associated with other disease conditions (Table 1). Other than genetic factors, people also develop HHcy via various environmental factors, including consuming excess amounts of a methionine-rich diet, vitamin B12/folate deficiency, alcohol intake, diabetes, and the obstruction of renal clearance [46,47]. Under HHcy conditions, the methionine cycle is generally dysregulated [48], so this leads to the disruption of multiple signaling pathways because it is the only pathway that gives rise to the production of essential methyl groups needed for the subsequent biosynthesis of cellular compounds (for example, creatine, epinephrine, carnitine, phospholipids, proteins, and polyamines) and also methylation of DNA, RNA, and histones [8,49].

## 3. Homocysteine Metabolism in Cancer

Hcy metabolism depends on several factors, including the intake of methionine in the diet, the production of SAM, and the type of cells in which methionine metabolism occurs [70]. Previous studies showed that high SAM levels can act as an allosteric inhibitor of methylenetetrahydrofolate reductase (MTHFR) [17]. MTHFR enzymes convert the 5,10-MTHF to 5-MTHF in the remethylation reaction [71]. So, high SAM levels prevent Hcy from entering the remethylation pathway. Interestingly a high SAM level also acts as an allosteric activator for the CBS, a rate-limiting enzyme of the transsulfuration pathway [72,73]. This suggests that high SAM levels favor Hcy entering the transsulfuration pathway. Cancer cells depend on the methionine cycle for their methylation reaction, producing high SAM, which leads to more production of GSH and H_2_S via the transsulfuration pathway. Hcy metabolism also depends on the dietary methionine load, which can affect SAM synthesis, suggesting a link between diet and cancer risk [10,74,75,76]. Studies suggest that when our diet contains a basal methionine level, Hcy goes to the remethylation pathway about 1.5–2.0 times more than the transsulfuration pathway [77]. Alternatively, when we take high methionine levels from the diet, Hcy cycling through remethylation is reduced by about 1.5-fold [77]. HHcy is found in ~5–7% of the general population and is associated with other disorders [7], including cancer [8,78,79,80,81]. Hcy metabolism pathways, including the transsulfuration and remethylation pathways, are associated with several types of cancer [82,83,84,85,86,87,88,89,90,91]. Recent advancements in research found a close link between HHcy and cancer that is discussed in the following paragraph.

## 4. Association of Hyperhomocysteinemia and Cancer

A study by Lily L Wu and James T Wu showed that patients (who were not taking antifolate drugs) with breast, ovarian and pancreatic carcinoma had elevated serum Hcy levels [92]. Elevated Hcy is also associated with a rapid proliferation rate of tumors in leukemia patients [93] and ovarian cancer [94]. Cancer cells have a high proliferation rate, so they depend more on the methionine cycle for the DNA, RNA, and histone methylation reactions. This methionine dependency or overproduction of Hcy could be a phenotypic expression of malignancy. This suggests that elevated Hcy could be an early carcinogenesis marker and a sensitive marker for detecting recurrence. Serum tumor markers have been used most frequently for monitoring cancer patients during therapy [95].

### 4.1. High Plasma Hcy Levels and Cancer

High homocysteine levels have been associated with various types of cancer, as summarized in Table 2. These studies (in Table 2) suggest that patients with advanced-stage cancer show higher Hcy levels than patients with early-stage cancer. This suggests that high Hcy levels can lead to apoptosis, and cancer cells in the late stage are more proliferative, so they secrete Hcy outside the cells.

Additionally, patients who underwent surgery or chemotherapy showed increased Hcy levels in their blood. As most of the chemotherapy drugs (alkylating agents, antimetabolites, methotrexate, hormones, and antagonists) are antifolate, folate deficiency can increase Hcy levels in these patients [119]. Another study showed that older cancer patients have a higher risk of developing HHcy than younger [120], suggesting age is another causing factor for high Hcy levels in cancer patients. Venous thromboembolism (VTE) is the most common complication associated with cancer, and it is also shown to be the most common cause of death in cancer. Interestingly, HHcy patients also developed venous thromboembolism, suggesting a link between cancer-associated complications and high Hcy levels. Moreover, a study showed that cancer patients without HHcy did not show venous thromboembolism [121].

### 4.2. Alteration in Hcy Metabolism Gene and Risk of Cancer

Previous studies have identified numerous enzyme mutations and polymorphisms (*MTHFR*, *CBS*, *MTRR*, *MTR*, *MTHFD*, *BHMT*, *TYMS*, *TCN 2*) that regulate Hcy metabolism [122,123,124,125,126,127,128]. These mutations and polymorphisms are often linked to HHcy and different cancer types (Table 2). The most common mutations in MTHFR 677C->T transition at codon 222 and 1298A->C transversion at codon 429 have been associated with cervical [68], colorectal [129], endometrial [130], and esophageal cancer [131]. Interestingly, the 677TT and 1298CC homozygotes have been found to have reduced prostate cancer risk, as the frequencies are very low (9 and 11%, respectively) [90,99], suggesting the risk factor of specific polymorphism depends on the types of cancer. In addition to genetic polymorphisms, many environmental factors, including folate status, methionine, and the effects of alcohol consumption, play a vital role in the causation of cancer. This understanding gives rise to the targeted therapy approach, where specific mutation types can be targeted with a specific drug. Similarly, *MTRR* gene A66G Ile22Met is found to be associated with colorectal cancer [87] and leukemia [21]. It is also noted that homozygotes (GG) have a three-fold higher risk of colorectal cancer than with that heterozygote (AG) polymorphism. As this allelic frequency varies between the different ethnic groups, this suggests that some populations may have a higher risk for certain types than others. Likewise, one significant polymorphism (*MTR* A2756G; Asp919Gly) has been documented in *MTR* [28]. A 1958G->A; Ala653Gly polymorphism in the *MTHFD*-1 gene was associated with acute lymphoid leukemia [132], but no association was reported with lung cancer [133]; an inverse association was reported with colon cancer.

### 4.3. Homocysteine-Mediated Epigenetic Alterations and Risk of Cancer

Epigenetics are the process of changes in phenotype without alteration of the DNA sequence; this can be heritable or achieved through gene–environmental interaction [134]. There are three types of epigenetic modification: (1) DNA methylation, (2) histone modification, and (3) RNA interference. Methylation can occur in DNA, RNA, and histone protein, and this process is mediated via the methionine cycle. There are three DNA methyltransferase (DNMT) types: DNMT1, DNMT3a, and DNMT3b. SAMs act as a crucial substrate methylation reaction via DNMTs. SAM levels can be changed via environmental factors like a high methionine diet, folate deficiency, vitamin B6, and vitamin B12. Many studies have connected Global DNA hypomethylation to cancer [135], suggesting that cancer cells show differential signaling than normal cells due to high SAM levels.

Gene activation or deactivation depends upon the methylation pattern of the N-terminal tail of histones [136]. Moreover, crosstalk between these histone tail modifications (methylation, acetylation, and homocysteinylation) may have mechanistic linkages with different types of cancer [137]. Although many studies showed that high Hcy levels are associated with different epigenetic alterations and associated with cellular pathology [8,138], minimal studies have shown the role of these modifications in cancer. A study noted that Hcy in various concentrations might alter gene silencing and activation in different patterns [139]. Studies suggest that severe HHcy may induce more injurious effects via alteration of the methylation reaction [140].

Global genomic hypomethylation has been found in many types of cancer, including metastatic prostate, chronic lymphocytic, and hepatocellular carcinoma [141,142,143,144]. Regional hypomethylation of DNA sequences is also often observed during the early stages of tumorigenesis and in abnormal nonneoplastic tissue, such as hyperplasia [145]. DNA hypomethylation leads to the decondensation of pericentromeric heterochromatin and the activation of retrotransposon elements [146]; these have been associated with activating some oncogene and deactivating some tumor suppressor genes [147].

#### 4.3.1. Hcy-Mediated DNA Methylation and Cancer

A previous study showed that methionine-rich food induces intra-cellular SAM levels, and as a consequence, global hypermethylation occurs and induces Hcy levels [148]. Where another study noted elevated Hcy-induced SAH levels, this induced SAH, in turn, inhibited SAM-dependent methyltransferases (such as DNMTs) via a negative feedback mechanism [140]. These studies suggest high Hcy levels may result in DNA hyper/hypomethylation. Moreover, many researchers using human and animal models proposed that HHcy leads to hyper/hypomethylation in a tissue-specific manner [149,150,151]. Cancer patients often show high Hcy levels, suggesting a possible link between Hcy-mediated hyper/hypomethylation and the causation of different types of cancer. Indeed, a study found HHcy-mediated hypermethylation of CpG islands located in the promoter of the ERα gene in breast cancer cell cells [152]. Interestingly, Zhang et al. showed that 10 and 30 μmol/L Hcy levels induced hypomethylation, whereas 100 and 300 μmol/L Hcy levels induced hypermethylation in the promoter of the Dimethylarginine Dimethylaminohydrolase 2 (DDAH2) gene [153]; this result suggests that hyper/hypomethylation may also depend on levels of Hcy production. Additionally, the methylation pattern also depends on many other factors such as DNA replication, chromatin accessibility, local availability of SAM, nutritional factors (folate supplementation), and aging [154]. Although hypo/hypermethylation of DNA depends on the HHcy state and tissue types [62,149,150,151], very limited studies have been carried out so far to show the association of HHcy and causation, progression and metastasis of cancer.

#### 4.3.2. Hcy-Mediated Histone Modification and Cancer

Histone protein is present in the nucleosomes, where DNA molecules warp around at specific intervals [155]. Many post-translation modifications (acetylation, methylation, phosphorylation, ubiquitination, and sumoylation) of histones lead to gene activation and inactivation [156]. These modifications are dynamic; one set of enzymes (called writers) can put down these activation/repressive marks, and another group of enzymes (erasers) can reverse these marks [156]. Although alteration of histone modifications can cause upregulation or downregulation of specific gene expression, minimal studies have been conducted on HHcy-mediated histone modification and its associated pathology in cancer. Since HHcy can inhibit SAM-dependent methyltransferases via a negative feedback mechanism [157], it can be concluded that HHcy can also alter histone methylation patterns that might influence tumor formations. Indeed, some studies found that these histone modifications act as drivers for different types of cancer, as reviewed by Levi A Garraway et al. and Kristian Helin et al. [158,159]. However, histone modifications also vary between cell types [160], so various histone modifications may lead to different types of cancer. Which factors and how these modifications have been regulated in different cell types that lead to different types of cancers is something that needs to be explored in the near future.

#### 4.3.3. Hcy-Mediated RNA Interference and Cancer

Earlier researchers used to think that RNA had only a housekeeping function (tRNAs and rRNAs) and a messenger function (mRNA) [161]; however, recent studies have found many new classes of regulatory non-coding RNAs. Some important non-coding RNAs are micro-RNA, endogenous small interfering RNAs (endo-siRNAs), PIWI-associated RNAs (piRNAs), and long non-coding RNAs. The discovery of non-coding RNAs has completely updated our understanding of cancer research [162]. The prognosis value of microRNA (miRNA) and long non-coding RNA (lncRNA) are widely reported in cancers [163,164]. Many studies showed that HHcy interferes with microRNA regulation and long non-coding RNA (lncRNA) [165], suggesting a link between HHcy and abnormal gene expression in cancer progression. Although most cancer research has focused on the abnormal expression of oncogenes or tumor suppressor genes, 97% of the human genome consists of non-coding sequences, leading researchers to investigate this dark matter of tumorigenesis. Non-coding RNAs can induce tumorigenesis and tumor progression via transcriptional and post-transcriptional modification, chromatin remodeling, and signal transduction. Although, to date, most of the integration of non-coding RNAs and tumorigenesis is still unknown, current research has started uncovering the complex network of the interaction of non-coding RNAs and how they modify the expression of oncogenes and tumor suppressor genes. These non-coding RNAs present in a tissue-specific manner and are considered as diagnostic, prognostic, and therapeutic targets in different diseases. There is growing research about the dysregulation of Circular RNAs (circRNAs) in cancer [166,167,168,169]. Recent reports show that circRNAs play essential roles in prostate cancer’s progression, proliferation, and epithelial–mesenchymal transition (EMT) [170]. In our previous studies, we noticed that under HHcy conditions circRNAs profile differently than in normal conditions [166,167,171]. HHcy-mediated, non-coding RNAs vary in different tissue types, suggesting more research is needed to identify specific changes in non-coding RNA based on the cancer types.

### 4.4. Hcy-Mediated H_2_S Production and Risk of Cancer

H_2_S was previously thought of as a toxic gas. However, recent studies found that other than from gut microbiota, H_2_S is produced inside a cell via Hcy metabolism [172]. H_2_S acts as a gasotransmitter like other gasotransmitters (for example, nitric oxide) and has a cytoprotective role [17]. H_2_S plays a crucial role in reducing oxidative and ER stress during HHcy conditions [7], suggesting a favorable role of H_2_S in cancer progression. Many studies also reported the role of H_2_S in cell proliferation, viability, and migration of cancer cells [173]. The CBS gene in Hcy metabolism typically catalyzes the condensation of serine with Hcy to produce cystathionine (in a transsulfuration reaction), whereas it produces H_2_S via β-elimination and β-replacement reactions [174]. Both β-elimination (catalysis of cysteine) and β-replacement (reaction of L-cysteine and 2-mercaptoethanol) reactions produce H_2_S. Many clinical studies have shown that there is *CBS* overexpression and increased H_2_S production in many cancer types [175,176]. Previous studies showed that tumor cells have a high proliferative rate, producing a massive amount of reactive oxygen species (ROS) [177] and needing neoangiogenesis [178]. In contrast, many studies have suggested that H2S reduces oxidative stress, induces cell proliferation and viability, and improves neoangiogenesis [7,48,172,179]. As SAM is an allosteric activator of CBS that binds to the regulatory domain of *CBS* and regulates H_2_S production, indeed, it helps in the growth of tumor cells [180]. Therefore, future strategies to treat cancer patients should involve modulation of CBS and H_2_S levels.

## 5. Multifactorial Role of H_2_S in Cancer

**Figure 3:**
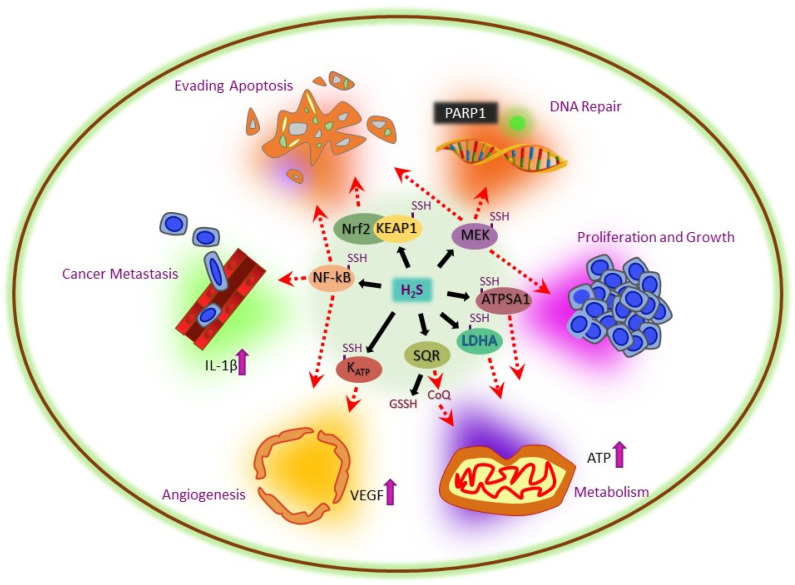
Different signaling pathways showing the multifactorial effects of H_2_S in cancer development. The cartoons represent the six cancer hallmarks influenced by H_2_S/H_2_S-mediated protein persulfidation.

Recent studies showed that H_2_S production helps induce cancer cell proliferation, viability, invasion, and metastasis [18]. Increasing levels of H_2_S have been proposed to induce cancer development by regulating a wide variety of cancer-related processes; this suggests that targeting H_2_S production could be a beneficial tool for cancer treatment. This section focuses on how H_2_S plays a role in cancer progression by targeting different processes, including oxidative stress, anti-apoptosis, DNA repair, tumor growth, cancer metabolism, metastasis, and angiogenesis (summarized in Figure 3).

### 5.1. H_2_S Production via Dysregulation of CBS, CSE, and 3MST Genes in Cancer

**Figure 4:**
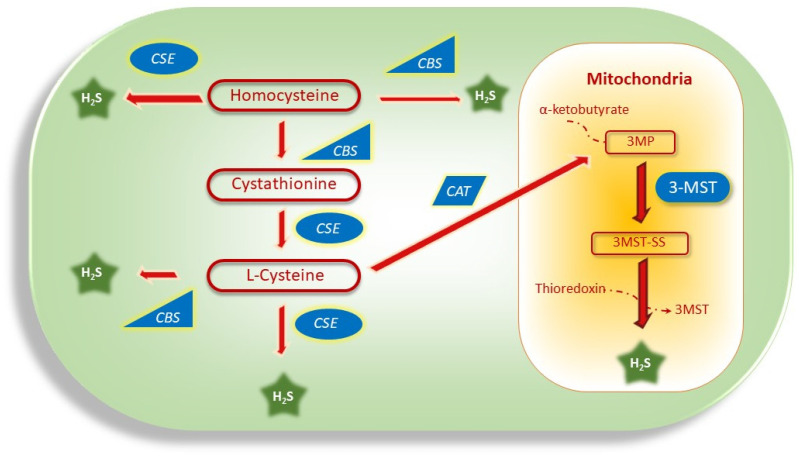
Signaling pathways of H_2_S production via Hcy metabolism. In the cytoplasm, H_2_S is produced from Hcy with the help of the CBS and CSE enzymes, whereas in mitochondria, H_2_S is produced with the help of the 3-MST enzyme.

H_2_S is produced endogenously through the transsulfuration pathway (involving CBS, CSE, and 3MST enzymes) of Hcy metabolism, as shown in Figure 4 [181]. Three enzymes that catalyze H_2_S production are often found dysregulated in cancer, as shown in Table 3.

#### 5.1.1. Dysregulation of CBS in Cancer

The main rate-limiting enzyme of the trans-sulphuration reaction of Hcy-metabolism is CBS, which catalyzes H_2_S production by driving the beta-replacement. The CBS gene is often found to be upregulated in colon cancer, ovarian cancer, breast cancer, thyroid cancer, and gallbladder adenocarcinoma tissues [182,184,185,197]. A study found that DNA methylation of the CBS promoter favors colon cancer progression [198]. SAM can allosterically activate the CBS gene to favor the cell proliferation of colon cancer cells [199]. Additionally, CBS can also be controlled via its redox sensitivity through the ^272^CXXC^275^ motif [200]. The high proliferation of cancer cells creates redox stress conditions inside the cells, which activates the CBS gene to produce H_2_S through the ^272^CXXC^275^ motif [200]. Although some studies showed that CBS expression is downregulated in glioma tumor cells, gastrointestinal cancer cells [186,187,201], and hepatocellular carcinoma, alternatively, reduced CBS expression upregulates the 3-MST gene in glioma tumor cells [202].

#### 5.1.2. Dysregulation of CSE in Cancer

CSE is one of the three H_2_S-producing enzymes in the transsulfuration pathway of Hcy metabolism. CSE has been upregulated in multiple cancer types, including prostate cancer, gastric cancer, and melanoma cells [190,203,204]. CSE was found to be induced by oxidative stress, ER stress, Golgi stress, inflammation, and starvation [205]. Unlike CBS, CSE can be upregulated transcriptionally via cellular stress response [206]. Under oxidative stress condition, nuclear factor (erythroid-derived 2)-like 2 (Nrf2) induces CSE expression through binding to its antioxidant response element (ARE) at 5′-untranslated regions (UTR), which in turn induce H_2_S production [207]. Overexpression of another transcription factor, specificity protein (SP) 1, induces H_2_S generation via binding to the CSE promoter [208]. Similarly, another study showed that tumor necrosis factor α (TNFα) induces H_2_S production through SP1-mediated CSE promoter binding [209]. In prostate cancer, a study found that CSE over-expression increased H_2_S production that led to the activation of nuclear factor-κB (NF-κB)-mediated interleukin 1β (IL-1β) signaling, resulting in enhanced cell invasion, angiogenesis, lymphangiogenesis, tumor growth, and metastasis [210]. Moreover, the upregulation of CSE by the STAT3 pathway increased breast cancer cell proliferation, growth, and migration [188]. Similarly, the upregulation of CSE by the Wnt/β-catenin pathway increased cell proliferation in colon cancer [193] whereas by extracellular signal-regulated protein kinase 1/2 (ERK1/2) pathway increased cell proliferation in liver cancer [192].

#### 5.1.3. Dysregulation of 3MST in Cancer

3MST is another H_2_S-producing enzyme primarily regulated through a redox-sensitive mechanism [206]. As oxidative stress is one of the characteristics of cancer cells, it seems that cancer cells primarily depend on 3MST for H_2_S production. During oxidative stress condition, 3MST becomes activated via oxidation at Cys^247^ and subsequently produces H_2_S to control cellular homeostasis [211]. Indeed, pharmacological inhibition of 3MST has been found to reduce cell proliferation, migration, and bioenergetics in colon cancer cells [212]. More research is needed to understand how different cancer types regulate 3MST to maintain their cellular redox balance.

### 5.2. H_2_S-Mediated Redox Balance in Cancer

As cancer cells have a high proliferative rate, they produce many free radicals [213]. There is the possibility that cancer cells upregulate H_2_S-producing enzymes in order to handle oxidative stress. Multiple studies demonstrated the cytoprotective effects of H_2_S in different in vitro models, all relating to its ability to neutralize a variety of reactive species [214,215,216] and reduction of a disulfide bond in proteins [217,218]. H_2_S in water dissociates into H^+^, HS^−^, and S^2−^ ions. HS^−^ has the capacity to scavenge ROS. H_2_S itself has also been recognized to be a reducing agent, as it can react directly with and quench the superoxide anion (O^2−^) [219,220] and free radicals like peroxynitrite [221] as well as other ROS in vitro. Micro concentrations of H_2_S generated from Na_2_S/NaHS were found to neutralize free oxyradicals [222], peroxynitrite [214], hypochlorous acid [215], and Hcy [216] in in vitro conditions. There is no sulfide receptor in mammalian cells that is responsible for the biological actions of sulfide; hence sulfide, as a thiol with strong reducing activities, may also be a redox-controlling molecule similar to other small thiols, such as cysteine and GSH [214,223]. A study using primary cultures of neurons found that H_2_S increases cellular GSH levels by enhancing gamma-glutamylcysteine synthetase activity and upregulating cystine transport [223]. Similarly, another study reported that 100 μM NaHS induces glutamate uptake by assisting glial glutamate transporter-1 (GLT-1) and enhances cysteine transport and GSH synthesis [224]. In support of this effect, multiple studies demonstrated that H_2_S induces cellular GSH in the brain [225], spinal cord [226], heart [227], lung [228], kidney [229], liver [228], and gastrointestinal tract [230,231]. Moreover, recent reports suggested that H_2_S could attenuate cellular oxidative stress by improving the activities of catalase [227,232,233,234] and glutathione peroxidase [235,236,237].

### 5.3. H_2_S-Mediated Recovery of Hypoxia in Cancer

Hypoxia is one of the hallmarks of solid tumors. H_2_S has been widely studied for its effects on the regulation of oxygen homeostasis via inhibiting HIF-1α activation [238]. Different studies found upregulation of H_2_S-producing enzymes under hypoxia conditions and its associated cancer progression [239,240]. In addition to this, our previous studies found that under hypoxia conditions, H_2_S induces neoangiogenesis via upregulation of the PPAR-c/HIF-1α signaling pathway [48]. Similarly, another study identified that H_2_S enhances HIF-1α expression via the downregulation of miR-640 [241]. In non-small cell lung cancer, a study proposed that H_2_S might activate HIF-1α via the PI3K/AKT pathway leading to angiogenesis [242]. Similarly, another study showed that under hypoxia, cancer cells produce H_2_S via induction of CSE to facilitate angiogenesis [243].

### 5.4. H_2_S-Mediated Recovery of Apoptosis in Cancer

Apoptosis is the process of cell death that happens naturally due to physiological or environmental stress [2]. Inhibition of apoptosis is one of the hallmarks of cancer progression that allows cancer cells to survive under various stresses [244]. Recent studies found that H_2_S has an antiapoptotic property in various cell types [17]. Different studies also found that cancer cells produce more H_2_S to evade apoptosis [245,246,247,248]. These studies suggest that, like classical antioxidants (for example, GSH), H_2_S inhibits apoptosis in cancer cells via scavenging ROS and reactive nitrogen species (RNS). The cancer cell has a high metabolic activity due to the high proliferative rate, and this leads to the generation of ROS and RNS. So, to recover from this oxidative stress condition, cancer cells need to produce more antioxidants like H_2_S to create profound antioxidant protection [206].

H_2_S not only suppresses apoptosis through the reduction in oxidative stress but is also found to activate various antiapoptotic pathways, including NF-κB [209], kelch-like ECH-associated protein 1 (Keap1) [249], and mitogen-activated protein kinase kinase 1 (MEK1) [250]. When NF-κB signaling becomes activated, it further activates multiple antiapoptotic genes, including the X-linked inhibitor of apoptosis protein (XIAP), cellular Inhibitors of Apoptosis Proteins (cIAPs), and the B-cell lymphoma 2 (Bcl-2) [251]. In contrast, Keap1 is mediated by persulfidation by H_2_S; after persulfidation, Keap1 acts as an adaptor for the Keap1-Cul3-RBX1 E3 ligase complex, which targets Nrf2 to proteasomal degradation [252]. Nrf2 acts as a transcription factor for genes containing antioxidant response elements (AREs) to suppress apoptosis in cancer cells [252]. The other process of H_2_S-mediated inhibition of apoptosis is via the activation of MEK1, which is one of the classical MAP kinase family proteins. MEK1 generally suppresses apoptosis via inhibition of the expressions of apoptotic-related proteins, including Bad, Bim-EL, Caspase 9, MCL-1, and TNFR [253].

### 5.5. H_2_S-Mediated DNA Repair in Cancer

H_2_S has been found to activate the DNA repair process via MEK1/Protein poly [ADP-ribose] polymerase 1 (PARP1)-mediated signaling pathways in cancer cells [250]. After the persulfidation of MEK1 at Cys^341^ residue by H_2_S, MEK1 translocates to the nucleus to stimulate PARP-1. PARP-1 is widely known as a sensor of DNA single- or double-strand breaks [254]. This suggests that cancer cells may use H_2_S to recover from DNA damage during proliferation. Moreover, H_2_S has also been found to help in mitochondrial DNA (mtDNA) repair via persulfidation on mt-specific DNA repair enzymes EXOG at Cys^76^ [255], which suggests that cancer cells may skip the apoptosis process via H_2_S-mediated recovery of DNA damage.

### 5.6. H_2_S-Mediated Tumor Growth and Metastasis in Cancer

Different studies found that higher levels of H_2_S in multiple cancer types [182,188,210,212] and inhibition of H_2_S production via suppression of CBS or CSE activities cause a reduction in tumor growth in multiple cancer types [182,210,255]. This suggests the critical role of H_2_S in the growth, proliferation, and survival of cancer cells. In addition, many studies found that endogenous H_2_S promotes cancer cell migration and invasion in multiple cancer types [210,242,256,257]. These studies showed that H_2_S promotes the metastasis process via various mechanisms, which include induction of epithelial-to-mesenchymal transition (EMT). Moreover, NF-κB is a key player in cancer metastasis; as H_2_S induces the persulfidation of NF-κB, it helps p65 to translocate into the nucleus and induce expressions of the metastatic promoting gene [210].

### 5.7. H_2_S-Mediated Metabolism in Cancer

Cancer cells have a very high proliferative rate, so they require more ATP production to maintain cellular energetics [258]. Endogenous H_2_S production was shown to act as a metabolic substrate for mitochondrial ATP production in cancer cells [199]. Moreover, H_2_S was found to increase the catalytic activity of mitochondria ATP synthase via persulfidation of ATP synthase (ATP5A1), which may induce mitochondrial ATP production [258]. To support their high growth rates, cancer cells preferentially convert glucose to lactate by aerobic glycolysis even in sufficient O_2_ (Warburg effect). In this process, lactate dehydrogenase A (LDHA) acts as a key player, and it is found that cancer cells induce LDHA activity via the persulfidation of LDHA at Cys^163^. Consistent with this, depletion of H_2_S production via knockdown of CBS was also found to reduce ATP production in cancer cells [176,182].

### 5.8. H_2_S-Mediated Angiogenesis in Cancer

Angiogenesis is one of the hallmarks of cancer; during tumor growth and metastasis, tumor cells secret proangiogenic factors such as VEGF [259]. Previous studies found that H_2_S induced angiogenesis under different disease conditions [48], including cancer [243]. Similarly, suppressing H_2_S production via silencing the CBS gene reduced angiogenesis in colon and ovarian cancer [176,182]. Additionally, suppression of H_2_S production via silencing another H_2_S-producing enzyme, CSE, was found to block angiogenesis [210]. Moreover, H_2_S was found to promote angiogenesis via activation of HIF-1α [197]. Additionally, H_2_S-mediated induction of angiogenesis has been found via NF-κB/IL-1β, PI3K/AKT, and MAPK signaling pathways [199,210].

### 5.9. H_2_S-Mediated Reduction in ER Stress in Cancer

As cancer cells have a high proliferation rate, they create different gene mutations, produce more misfolded proteins, and induce ER stress response [260]. As H_2_S was found to reduce ER stress in different disease conditions [7,261,262,263], it suggests that cancer cells may produce more H_2_S to recover from ER stress. In addition, as cancer cells mainly depend on the methylation cycle, this also produces a high amount of Hcy, which induces homocysteinylation of protein and further activates ER stress [264]. There is also the possibility that H_2_S can reverse protein homocysteinylation [265].

## 6. Current Cancer Therapeutics Targeting the Hcy and H_2_S Signaling and Their Limitations

Current treatment options for cancer are based on specific types of cancer and the stage of cancer; these include chemotherapy, radiation therapy, immunotherapy, and targeted therapy [266]. While treatment increases the lifespan of many patients, it is also associated with many side effects that will determine the health consequences. Also, the efficacy of these treatment options is limited by the resistance that patients develop [267]. As chemotherapy has many side effects due to the property of killing normal healthy cells, recent treatment is shifting gears toward targeted therapy approaches.

Methionine is an essential amino acid, and many tumor cells show dependence on exogenous sources of methionine [4]. Studies showed that methionine restriction inhibits cancer cell growth proliferation while normal cells remain unaffected [4]. In addition, methionine restriction showed enhanced efficiency of chemotherapy and radiotherapy in animal models [268]. A previous study showed that methionine restriction for an average of 17 weeks is safe and feasible in patients with advanced metastatic cancer [14].

Moreover, there have been many drugs developed that target the methionine cycle, but none of them showed clinical success. Antifolate drugs (for example, methotrexate) that interfere with the folate cycle have shown limited clinical efficiency due to side effects and resistance [269,270]. As the methylation cycle is essential for normal cells, these drugs kill both cancer and healthy cells. However, small molecule inhibitors that target serine synthesis pathways have been successful in in vitro and animal studies [271,272]. However, in order to reduce side effects, the drug used has to be specific to the particular cancer. For small molecule inhibitors that inhibit PHGDH (for example, NCT-503, CBR-5884), the tumor has to be fully addicted to PHGDH. Also, it needs to be ensured that this drug does not interfere with any other signaling pathways critical for signaling. If any type of tumor is not fully dependent on a specific pathway, that means the cancer cells may be using another source for that specific pathway. For targeting the serine synthesis pathway, if any specific cancer cell lines do not respond to the drug, these cells may be using exogenous serine supplementation. So, to target this cancer type with this drug, we should also consider the external source of nutrients/diet. Another type of mechanism is called the compensatory mechanism, by which cancer cells become resistant to specific drugs. For example, any drug targeting mitochondrial methylenetetrahydrofolate dehydrogenase (MTHFD1L) cancer cells can compensate using cytoplasmic MTHFD1 [273].

There are variable reports so far documented when it comes to targeting H_2_S metabolism. Lower doses of H_2_S donor compounds were found to have pro-cancer activity by different mechanisms [274], whereas higher doses had anticancer activity due to uncontrolled intracellular acidification [275,276,277]. Various H_2_S donor compounds (for example, NaHS, Na_2_S, GYY4137) tested preclinically for their anticancer property [275,276,277]. In contrast, as many studies showed, endogenous H_2_S has beneficial effects for tumor growth and metastasis, and inhibiting endogenous production of H_2_S (via targeting H_2_S-producing enzymes) may be a good strategy. DL-propargylglycine (PAG) is an inhibitor of CSE that showed limited cell permeability [218] and non-selective inhibition of other enzymes [278,279,280]. Another inhibitor of H_2_S-producing enzymes is aminooxy acetic acid (AOAA), which is also found to inhibit cysteine aminotransferase (CAT) [281]. HMPSNE is an inhibitor that targets the 3rd H_2_S-producing enzyme 3MST, showed the highest selectivity for 3MST [31,282], and was found to inhibit cell proliferation of colon cancer [212,283]. In order to make a more efficient drug that inhibits H_2_S production, more research is needed.

Although targeting H_2_S production showed promise, there are a few limitations. Firstly, many previous studies that targeted H_2_S production used the pharmacological inhibitor AOAA (a CBS inhibitor). However, AOAA showed nonspecific inhibition of CSE, 3MST, and over thirty other cellular enzymes [284]. Similarly, another H_2_S production inhibitor, β-cyano-alanine, showed suboptimum specificity [285]. Secondly, there are functional differences between H_2_S production enzymes in different cancer types. For example, in prostate cancer, mutant CSE was found to be lowly invasive but did not interfere with cell migration capacity [210], suggesting the mutation-specific targeting of H_2_S production will be necessary for future therapeutics.

### A Hypothesis of Targeting the Hcy and H_2_S Balance for Cancer Treatment and Its Application

**Figure 5:**
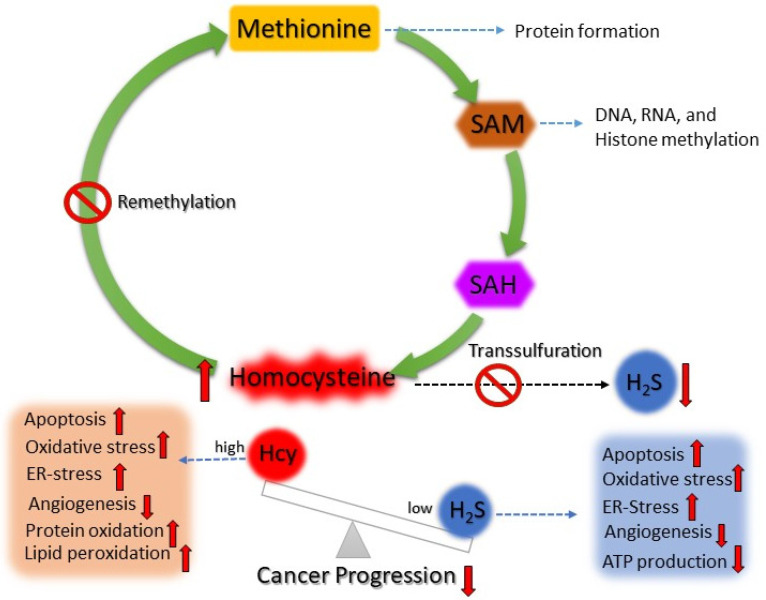
Cartoon diagram showing how homocysteine and H_2_S balance can be targeted for cancer treatment. Targeting the transsulfuration and remethylation pathway in cancer cells will build up highly toxic homocysteine inside the cells. Consequently, cancer progression will be inhibited via apoptosis, poor angiogenesis, protein oxidation, and oxidative and ER stress.

Due to the high proliferative rate, cancer cells require an external source of methionine for protein formation, methylation reaction (epigenetic alteration), and the production of H_2_S (antioxidant). When cancer cells use the methionine cycle, it produces a massive amount of Hcy, which is toxic for the growth of the tumors. As previous studies found that high SAM levels act as an allosteric inhibitor of MTHFR (involved in the folate cycle) and activator of CBS, so when cancer cells produce high SAM levels, it prevents Hcy from entering the remethylation pathway rather than allowing excess Hcy to shuttle to the transculturation pathway to produce H_2_S [71,72,73]. This production of high H_2_S in cancer cells helps them to survive, proliferate, grow, and metastasize. Although many therapeutic strategies have been developed either by methionine restriction or targeting different enzymes of the methionine cycle, folate cycle, and transsulfuration pathway, none of these treatment strategies showed effectiveness in clinical studies. So, in the future, more research is required where we can utilize their dependence on the methionine cycle and target specific enzymes to treat cancer. So far, we have noticed that cancer cells depend on the methionine cycle more, so if we can target both the transsulfuration pathway (via CBS) and the remethylation pathway at the same time, as shown in Figure 5, then these cells will build up toxic Hcy and inhibit the production of H_2_S. High levels of Hcy will induce apoptosis, protein oxidation, and oxidative and ER stress and inhibit angiogenesis, whereas low levels of H_2_S will inhibit cancer growths; as a result, tumor progression will be inhibited due to the effects of high Hcy and low levels of antioxidants like H_2_S.

Efforts to interfere with the methylation cycle in different cancers have reached a plateau, with only incrementally effective inhibitors developed to date. In order to overcome this barrier and develop highly effective inhibitors, we need to understand how to target this Hcy and H_2_S signaling more precisely with minimal side effects. Based on the above discussion, although it is apparent that targeting Hcy and H_2_S balance is beneficial for cancer treatment, there are however no studies have been carried out that exploit this circuit. This extensive review article will likely lead us and many in academia and industry to develop next-generation therapeutic agents targeting the blocking of H_2_S production and Hcy remethylation. The significance and impact would be profound in increasing efficacy and reducing toxicity for a large number of cancer patients where targeted therapy was shown to be non-effective. For example, in treating triple-negative breast cancer, this treatment strategy will be a good option as there is no other oncogene-driven monotherapy available. Similarly, cancer types that are more dependent on the methionine cycle will be the best option to use this strategy. Another exciting cancer treatment aspect that was not covered in this review is Hcy-mediated epigenetic alteration and H2S-mediated polysulfide production in cancer. So, a better understanding of their signaling in cancer will undoubtedly facilitate better treatment for cancer patients.

## 7. Conclusions

Given that cancer cells depend on the methionine cycle for their methylation reaction and H_2_S production, many researchers tried different strategies to target these signaling pathways. Unfortunately, none of the strategies turned out beneficial for cancer treatment. Based on current understanding, it is noted that indefinite targeting of the methionine cycle, either via a methionine restriction diet or targeting different enzymes of the methionine cycle, is not feasible due to the development of resistance, non-responsiveness, and numerous side effects. However, targeting H_2_S production showed to be somewhat promising based on its effects on cancer progression via inhibition apoptosis, oxidative stress, ER stress, and stimulation of the DNA repair process, cancer metabolism, tumor growth, and metastasis. Again, this strategy did not benefit cancer treatment due to nonspecific targeting. Therefore, for future prospects, it is necessary to target the transsulfuration pathway for blocking H_2_S production and the remethylation pathway to build up toxic Hcy. As we noticed, Hcy has detrimental effects on cells via apoptosis, protein oxidation, lipid peroxidation, poor angiogenesis, oxidative stress, and ER stress. So, excess Hcy build-up will not be able to recover via simultaneous blocking of H_2_S production, which leads to the regression of tumor growth. However, more research is anticipated to test this proof of concept for cancer treatment.

## Figures and Tables

**Table 1:** Association of Hyperhomocysteinemia with different disorders.

Genes	Polymorphisms	Condition	Associated Complications	References
*CBS*	844INS68	HHcy	Peripheral artery occlusive disease	[50]
	T833C	HHcy	Stroke	[51]
	844INS68	HHcy	Thrombosis	[52]
*MTHFR*	C677T	HHcy	Retinal vein occlusion	[53]
	C677T	HHcy	Stroke	[42,43,44,45]
	C677T	HHcy	Venous thromboembolism	[54]
	C677T	HHcy	Hypertension	[55,56,57]
	C677T	HHcy	Alzheimer’s Disease	[58]
	A1298C	HHcy	Cerebral venous sinus thrombosis	[59,60,61]
	C677T	HHcy	Hyperlipidemia	[62]
	C677T	HHcy	Diabetic nephropathy	[63,64,65,66]
	C677T	HHcy	Cerebral venous thrombosis	[67]
	C677T	HHcy	Parkinson’s Disease	[68,69]

**Table 2:** Association of polymorphisms homocysteine metabolism genes with cancer risk. (odds ratio is indicated as OR).

Genes	Polymorphisms	Cancer Types	Significant Association (OR)	References
*MTHFR*	677C-> T	Breast Cancer	Positive Association (1.19)	[22]
		Ovarian Cancer	No association (1.03)	[22]
		Esophageal Squamous Cell Carcinoma	Positive Association (1.47)	[96]
		Acute Lymphocytic Leukemia	Negative Association (0.99)	[21]
		Prostate Cancer	Negative association (0.78)	[23]
		Colorectal Adenomas	Negative association (0.76)	[97]
		Late-stage colorectal tumorigenesis	Positive Association (1.32)	[29]
		Endometrial Cancer	No association (1.10)	[98]
	1298A->C	Prostate Cancer	Negative Association (0.58)	[99]
		Acute Lymphocytic Leukemia	Negative Association (0.33)	[21]
		Acute Myeloid Leukemia	No association (1.00)	[88]
		Endometrial Cancer	No association (1.00)	[98]
*MTRR*	66A->G	Acute Myeloid Leukemia	Positive association for Asian population (1.40)	[100]
		Head and Neck Cancer	Positive Association (1.24)	[101]
		Colorectal Cancer	Positive Association (2.77, 1.15)	[87,102]
		Gastric Cancer	Positive Association (1.39)	[103]
		Breast Cancer	Positive Association (4.45)	[104]
*MTR*	b2756A->G	Colorectal Cancer	Positive Association (2.04)	[28]
		Primary Liver Cancer	No association (1.00)	[105]
		Breast Cancer	No association (1.00)	[106]
		Glioblastoma Multiforme	No association (1.00)	[107]
		Upper Gastrointestinal Tract cancer	No association (1.00)	[108]
		Digestive System Cancer	No association (1.00)	[109]
*MTHFD1*	1958G->A	Gastric Cancer	Positive Association (2.05)	[110]
	G1958A	Colon Cancer	Negative Association (0.89)	[111]
*BHMT*	742G->A	Head and Neck Squamous Cell Carcinoma	Positive Association (1.34)	[112]
		Breast Cancer	No association (0.98)	[113]
		Cervical Cancer	Negative Association (0.433)	[114]
		Ovarian Cancer	No association (1.00)	[115]
		Colorectal Adenoma	Positive Association (1.09)	[116]
*TCN 2*	776G>C	Glioblastoma Multiforme	No association (1.00)	[107]
		Primary Central Nervous System Lymphoma	No association (1.00)	[117]
*TYMS*	TS 3′-UTR	Esophageal and Stomach Cancer	No association (1.00)	[118]

**Table 3:** Association of different H_2_S-producing enzymes in different cancer types.

Enzymes	Cancer Types	Upregulation/Downregulation	Reference
CBS	Colon Cancer	Upregulation	[182]
	Ovarian Cancer	Upregulation	[176]
	Breast Cancer	Upregulation	[183]
	Thyroid Cancer	Upregulation	[184]
	Gallbladder Adenocarcinoma	Upregulation	[185]
	Hepatocellular Carcinoma	Downregulation	[186]
	Gastrointestinal Cancer	Downregulation	[187]
CSE	Breast Cancer	Upregulation	[188]
	Prostate Cancer	Upregulation	[189]
	Gastric Cancer	Upregulation	[190]
	Bladder Cancer	Upregulation	[191]
	Hepatoma	Upregulation	[192]
	Colon Cancer	Upregulation	[193]
	Renal Cell Carcinoma	Downregulation	[194]
3MST	Glioma Tumor	Upregulation	[195]
	Colon Cancer	Upregulation	[196]

## Abbreviations

SAH	S-Adenosyl Homocysteine
Hcy	Homocysteine
HHcy	Hyperhomocysteinemia
SAM	S-Adenosyl Methionine
H_2_S	Hydrogen Sulfide
GSH	Glutathione
ROS	Reactive Oxygen Species
ER	Endoplasmic Reticulum
MAT	Methionine Adenosyl Transferase
CBS	Cystathionine Β-Synthase
CSE	Cystathionine Γ-Lyase
MTHFR	Methylenetetrahydrofolate Reductase
MTRR	Methionine Synthase Reductase
MTR	Methionine Synthase
MTHFD	Methylenetetrahydrofolate Dehydrogenase
BHMT	Betaine Homocysteine Methyltransferase
TYMS	Thymidylate Synthase
TCN2	Transcobalamin 2
MTHFD1L	Methylenetetrahydrofolate dehydrogenase
DNMTS	DNA Methyltransferases
VSMCS	Vascular Smooth Muscle Cells
DDAH2	Dimethylarginine Dimethylaminohydrolase 2
HMT	Histone Methyltransferase
lncRNA	Long Non-Coding RNA
miRNA	MicroRNA
circRNA	CircularRNA
NF-κB	Nuclear factor kappa-light-chain-enhancer of activated B cells
Keap1	Kelch-like ECH-associated protein 1
MEK1	Mitogen-activated protein kinase kinase1
XIAP	X-linked inhibitor of apoptosis protein
cIAPs	Cellular Inhibitors of Apoptosis Proteins
Bcl-2	B-cell lymphoma 2 gene
AREs	Antioxidant Response Elements
